# Impact of an ADRD caregiver intervention during the Covid-19 pandemic

**DOI:** 10.1093/geroni/igaf122.674

**Published:** 2025-12-31

**Authors:** Lena Thompson, Freda Lynn, Emily Killian, Maria Donohoe, Hyunkeun Cho, Sato Ashida

**Affiliations:** University of Alaska Fairbanks, Fairbanks, Alaska, United States; University of Iowa, Iowa City, Iowa, United States; University of Iowa, Iowa City, Iowa, United States; University of Iowa, Iowa City, Iowa, United States; University of Iowa, Iowa City, Iowa, United States; University of Iowa, Iowa City, Iowa, United States

## Abstract

The Building a Bridge (BAB) intervention is a mixed-method clinical trial connecting caregivers with programs, resources, and information to support people newly diagnosed with dementia. BAB was forced to adapt during the Covid-19 pandemic—a time when services for people living with dementia (PLWD) and their caregivers were greatly reduced. This study assesses the experiences and impact of the pandemic-altered intervention to inform best practices for interventions that address caregiver needs during times of societal crisis. Participants completed baseline (n = 81) and 3-month follow-up (n = 53) telephone surveys (e.g., Zarit Burden; knowledge; self-efficacy). Qualitative data included participant responses to open-ended questions at 3-month follow-up and interventionists’ notes for 31 participants. Data were analyzed using change score regression models and rapid qualitative analysis. Statistically, intervention participants gained more knowledge about ADRD and caregiving compared to control (p=.036) and those with low knowledge at baseline gained more knowledge (p=.044). On average, self-efficacy did not statistically change and distress (e.g., burden, anxiety) increased. A sensitivity analysis confirms the robustness of results despite attrition. Participants described feeling more knowledgeable about dementia and future planning, and they consistently expressed gratitude for the interventionists’ emotional support. Interventionists reported providing unplanned support outside the intervention like support with selling a home. The BAB intervention did not increase self-efficacy to use services, likely because services were unavailable during the pandemic timeframe. However, it increased participants’ knowledge and connected them to social systems that allow them to activate resources when needed, building resilience to withstand challenging situations like the pandemic.

